# Systematic Review and Meta-Analysis of Human Studies to Support a Quantitative Recommendation for Whole Grain Intake in Relation to Type 2 Diabetes

**DOI:** 10.1371/journal.pone.0131377

**Published:** 2015-06-22

**Authors:** Aurelie Chanson-Rolle, Alexandra Meynier, François Aubin, Jenni Lappi, Kaisa Poutanen, Sophie Vinoy, Veronique Braesco

**Affiliations:** 1 VAB-nutrition, Clermont-Ferrand, France; 2 Mondelez France R&D SAS, Saclay, France; 3 Venn Life Sciences SAS, Paris, France; 4 Department of Clinical Nutrition, University of Eastern Finland, Kuopio, Finland; 5 VTT Technical Research Centre of Finland, Espoo, Finland; University of Tolima, COLOMBIA

## Abstract

**Background:**

Due to the increasing evidence of their health benefits, whole grains are recommended for consumption worldwide. Such recommendations are, however, rarely quantitative. Our aim was to perform a quantitative evaluation of the relationship between whole grain consumption and the occurrence of type 2 diabetes (T2D) to support a recommendation on the daily consumption of whole grains.

**Methods and Findings:**

We conducted a systematic review by searching three bibliographic databases. We included human studies addressing the relationship between whole grain consumption and T2D occurrence, and providing quantitative information on daily intake of whole grains. A dose-response meta-regression analysis between whole grain intake and T2D occurrence was performed, using a hierarchical mixed least square linear regression model. Eight observational studies were included (all but one prospective), with a total of 15,573 cases of T2D among 316,051 participants. Quantitative meta-regression demonstrated a significant linear inverse relationship between whole grain intake and T2D occurrence (*P*<0.0001), with an overall absolute reduction of 0.3% in the T2D rate for each additional 10 g of whole grain ingredient consumed daily. The association persisted when adjusted on sex, age, country, study design, follow up duration, and mode of report of whole grain intakes (as foods or ingredients).

**Conclusions:**

The meta-regression model made it possible to estimate the decrease in T2D risk corresponding to various changes in whole grain intakes, and the results contribute to setting up quantitative recommendations. For instance, consuming three servings of whole grain foods (45 g of whole grain ingredients) daily would induce a 20% relative reduction in the T2D risk as compared to consuming a half serving (7.5 g of whole grain ingredients). These results should be considered for future recommendations, by considering the actual whole grain intake of the concerned populations. The systematic review protocol was published on the PROSPERO register (CRD42013006925).

## Introduction

There is increasing evidence that consuming whole grains may reduce the risk of several chronic diseases. In particular, observational studies suggest that a diet rich in whole grain foods reduces the risks of type 2 diabetes (T2D) [1,2] and cardiovascular diseases [2,3], and has a beneficial impact on body weight [4] and mortality [5].

Several definitions are available for the term “whole grain,” all emphasizing the presence of endosperm, germ and bran in the same relative proportions as they exist in the intact kernel [6,7]. However, whole grains are rarely consumed alone and are most often ingredients within a food containing other components.

Recommendations on consumption of whole grain foods have been included in many dietary guidelines across the world. The recommendations vary widely depending on the country, from a generic message to increase grain consumption, preferably as whole grains, to a quantitative recommendation with a suggestion of whole grain amount [8,9]. Nevertheless, quantitative recommendations for the intake of whole grain ingredients that would give some practical guidance to consumers are rare today and vary widely from country to country (from 50 g/d in China to 115 g/d in the Netherlands) [9]. Such quantitative recommendations would however be important for Public Health purposes and should be based on the overall available evidence to support the beneficial health effects of whole grains in humans.

In most of the many meta-analyses performed on this topic, the objective has been to study whether or not an association exists between whole grain intake and health outcomes, and rarely to investigate which whole grain amount or which increase in the amount of whole grains appears to be effective. As an example, the recent meta-analysis of Ye *et al*. convincingly showed an inverse association between whole grain intake and the risks of T2D, cardiovascular diseases and weight gain; the authors compared the lowest versus the highest categories of whole grain intake within each identified study, which does not enable to estimate which amount of whole grains is the most likely to be effective [2]. De Munter *et al*. found a 21% reduction in the risk of T2D per 2 servings/d increment in whole grain intakes, using a semi-quantitative approach which did not allow assessing the dose-response effect of whole grains on T2D prevention [1].

The lack of a uniform definition of what constitutes a “whole grain food” is an additional challenge when attempting to consider whole grain amounts [8,9]. Jacobs *et al*. proposed a definition of whole grain foods as foods with ≥25% whole grain or bran content by weight [10]. In the United States, the Food and Drug Administration (FDA) approved a whole grain claim for foods that contain 51% or more whole grain ingredient(s) by weight [11]. Therefore, a given whole grain-containing food can be considered as a whole grain food in some studies, but not in others, leading to discordant estimations of whole grain intakes.

The aim of this work was to analyze the available scientific evidence in order to help derive a quantitative recommendation for the daily consumption of whole grains. For that purpose, we considered published human studies in which whole grain intakes could be reasonably quantified and we performed a systematic review and quantitative meta-analysis on the relationship between whole grain consumption and the following health outcomes: overall mortality, obesity, cardiovascular diseases, T2D and associated risk factors. This article describes the results related to T2D.

## Materials and Methods

The protocol of the systematic review has been published on the PROSPERO register (http://www.crd.york.ac.uk/prospero/) under registration number CRD42013006925 (see also S1 Protocol). This study complies with the requirement of the Moose statement [12] and the Moose checklist is available as supporting information (see S1 Checklist).

### Data sources and searches

A systematic search of three bibliographic databases [MEDLINE (http://www.ncbi.nlm.nih.gov/pubmed), Cochrane Central Register of Controlled Trials and Cochrane Database of Systematic Reviews (http://www.thecochranelibrary.com)] was performed covering the period from January 1^st^ 1993 to March 4^th^ 2015, by using the following search strategy: whole grain-related key words [e.g., “whole(-)cereal(s)” OR “whole(-)grains” OR individual cereal names such as “wheat” OR “rye”] were combined with key words related to the health outcomes of interest, namely, overall mortality, obesity, cardiovascular diseases, T2D and associated risk factors [e.g., “overall mortality” OR “obesity” OR “body weight” OR “cardio(-)vascular” OR “blood cholesterol” OR “diabetes” OR “insulin sensitivity”] (see S1 Fig). The search was limited to human studies and English language publications where limitation was possible (MEDLINE). In addition, the reference lists of included studies and previously published reviews were searched for additional potentially eligible studies.

### Study selection

**Fig 1:**
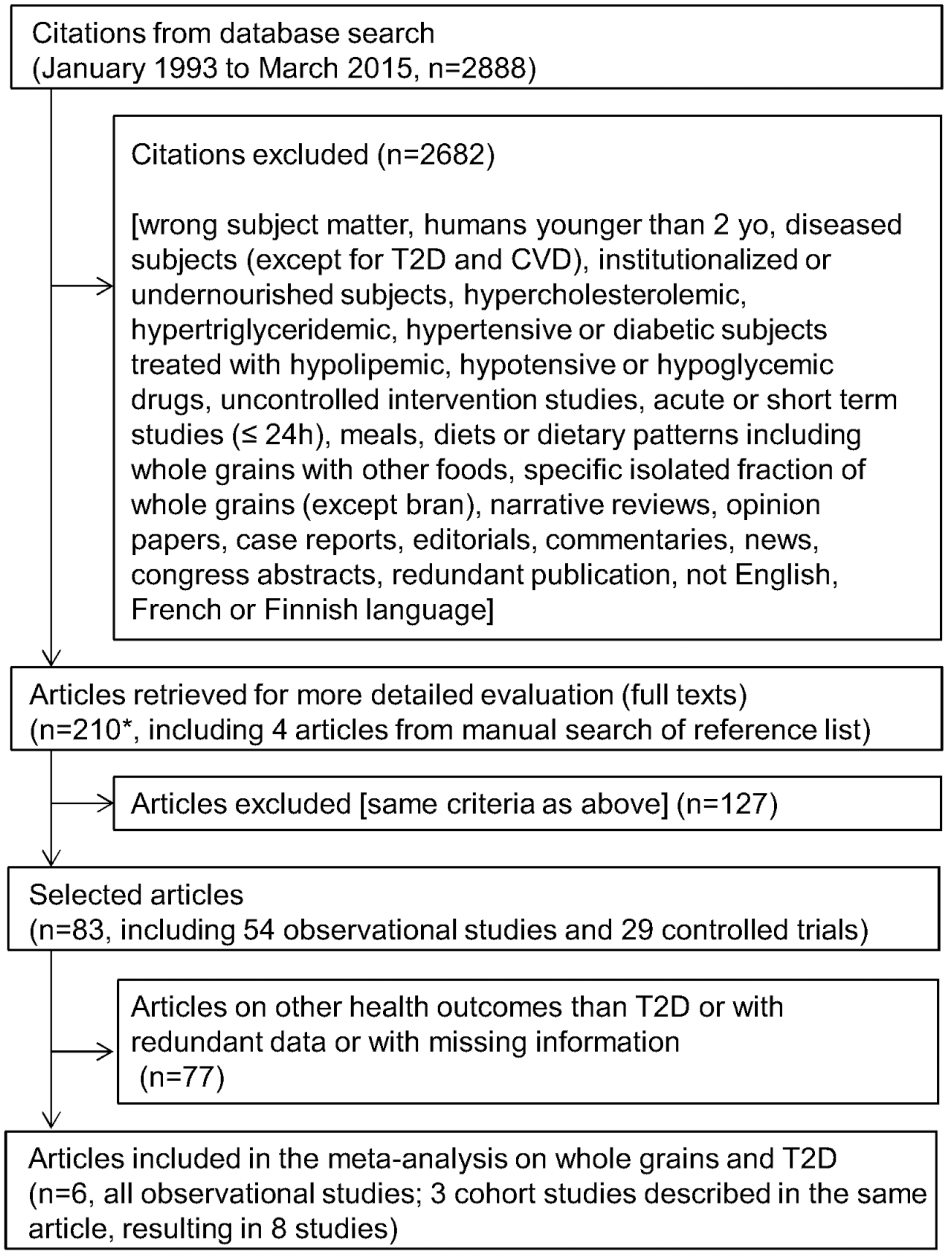
Flow diagram of study selection. * The list of the 210 articles selected for full text evaluation is available in S1 Table, which also describes the outcome of the selection process for each article (including justification for exclusion). CVD, cardiovascular diseases; T2D, type 2 diabetes.

Three reviewers (ACR, AM, JL) independently screened the titles and abstracts of all retrieved publications for inclusion or exclusion. Full text articles were obtained when abstracts were potentially relevant and were reviewed independently; conflicting views were resolved by discussions between the three reviewers. Studies were included in the systematic review if they were observational studies (including cross-sectional, case-control and cohort studies) or controlled interventional studies, addressing the relationship between whole grain consumption and one of the health outcomes of interest. They also had to provide quantitative information on daily intake of whole grains: information on whole grain consumption was given as intake categories of whole grain foods or whole grain ingredients in observational studies, or as consumption of whole grain foods or of diets high in whole grain foods in interventional studies. Studies carried out on the following populations were excluded: children younger than two years old; patients suffering from cancer, renal insufficiency, type 1 diabetes or other pathologies (to the exception of cardiovascular diseases and T2D); hypercholesterolemic, hypertriglyceridemic, hypertensive or diabetic subjects when treated with hypolipemic, hypotensive or hypoglycemic drugs; hospitalized or long-term institutionalized patients, and undernourished populations. Studies were excluded if they evaluated acute or short term effects of whole grain intake (e.g., postprandial studies or studies with a duration less than 24-h long), if they evaluated the impact of meals, diets or dietary patterns including whole grains with other foods, or if they evaluated a specific isolated fraction of whole grains (germ or aleurone), except bran. This process led to the selection of 83 studies (Fig 1).

For the purpose of the analysis presented here, the 83 selected studies were further screened to select only studies addressing the relationship between whole grain consumption and occurrence of T2D. In this case, only observational studies were identified (no interventional controlled trials were found). For a study to be selected, publication had to include, for each category of whole grain intake, quantitative information on daily intake of whole grains, number of subjects and occurrence of T2D (number of cases). Missing information was requested from authors. Studies were excluded if the needed information could not be obtained or calculated. When several publications dealt with the same cohort, only the publication with the longest duration of follow up was included.

### Data extraction

Data were extracted by three PhD (ACR, AM and VB) by using a predefined data template. Disagreements were resolved through discussion. Extracted data were as shown in column headings of Table 1. Furthermore, the following data were extracted, when available, for each category of whole grain intake: daily intake of whole grains (median, range or mean), number of subjects, occurrence of T2D (number of cases), number of person-years (total cumulative follow up), age (mean), sex (% males). The three reviewers independently evaluated the individual studies regarding the appropriateness of the sample size, of the description of inclusion and exclusion criteria and of baseline characteristics of the subjects, and of the methods used to address potential sources of bias and to adjust for the effects of potential confounding variables. Information on declaration of conflict of interest or identification of funding sources was also collected for individual studies.

**Table 1:** Characteristics of the eight studies included in the meta-regression analysis on whole grain intake and occurrence of type 2 diabetes.

Study (author, year)	Study name	Design	Country	Mode of report of WG intakes in publication^1^	Follow up (y)	Age range (y)	% males	N total	N T2D cases (%)	T2D assessment	Diet assessment	Method to categorize WG intakes	Median^2^ of WG daily intakes (lowest and highest categories) (g/d of WG ingredients)
Esmaillzadeh 2005 [18]	Tehran Lipid and Glucose Study	cross-sectional	Iran	Food (g/d)	NA	18–74	43	827	27 (3.3)	Biological diagnosis	FFQ	Quartiles	3.1 / 116.8 (mean)
Meyer 2000 [19]	Iowa Women's Health Study	cohort	USA	Food (servings / week)	6y	55–69	0	35988	1141 (3.2)	Self-report of physician diagnosis	FFQ	Quintiles	2.2 / 44.8
Montonen 2003 [20]	Finnish Mobile Clinic Health Examination Survey	cohort	Finland	Food (g/d)	19y	40–69	53	4316	156 (3.6)	National register confirmed by medical certificates	Diet history	Quartiles	40.3 / 154.0
Sun 2010 [21]	HPFS	cohort	USA	Ingredient (g/d)	20y	32–87	100	39765	2648 (6.7)	Confirmed self-report of physician diagnosis	FFQ	Quintiles	5.1 / 47.1
Sun 2010 [21]	NHS I	cohort	USA	Ingredient (g/d)	22y	37–65	0	69120	5500 (8.0)	Confirmed self-report of physician diagnosis	FFQ	Quintiles	3.6 / 31.3
Sun 2010 [21]	NHS II	cohort	USA	Ingredient (g/d)	14y	26–45	0	88343	2359 (2.7)	Confirmed self-report of physician diagnosis	FFQ	Quintiles	6.2 / 40.0
Parker 2013 [22]	WHI OS	cohort	USA	Food (serving/d)	8y	50–79	0	72215	3465 (4.8)	Self-report of physician diagnosis	FFQ	Arbitrary categories	2.8 / 39.6
Wirstrom 2013 [23]	NA	cohort	Sweden	Ingredient (g/d)	8-10y	35–56	42	5477	277 (5.1)	Diagnosis of diabetes (not further specified)	FFQ	Tertiles	20.7 / 76.5

^1^Information on how whole grain intakes were reported in the original publication (as amounts of whole grain foods or of whole grain ingredients, with the unit used indicated in brackets).

^2^Median values of lowest and highest categories of whole grain intakes, standardized in g/d of whole grain ingredients (except if indicated otherwise).

FFQ, food frequency questionnaire. HPFS, Health Professionals Follow-up Study. N, number of subjects. NA, not applicable. NHS, Nurses’ Health Study. T2D, type 2 diabetes. WG, whole grains. WHI OS: Women’s Health Initiative Observational Study. Y, years.

For the meta-analysis, all data on whole grain intakes were standardized in g/d of whole grain ingredients. Therefore, whole grain intakes which were given in amounts of whole grain foods in original publications were computed by assuming that one serving of whole grain foods was equivalent to 30 g, and that whole grain foods contained on average 51% of whole grain ingredients. The latter assumption was based on the FDA whole grain claim [11].

We were conscious that this assumption might be seen as a minimalist approach as many of the most consumed whole grain foods may contain more than 51% of whole grain ingredients [13]. However, regarding our ultimate objective of deriving a quantitative recommendation of whole grain intake for the population, we were concerned that it would be preferable to slightly underestimate the amount of whole grain which is associated to a beneficial health effect, leading to a slight overestimation of a possible recommendation, rather than the opposite, which may lead to abusively promote whole grain amounts which might be too low to elicit favorable health effects. The relevance of the 51% choice was further confirmed by the analysis of data on whole grain intakes from the United States which indicated that on average whole grain foods contained 54% of whole grain ingredients, by considering the percent of whole grain ingredients contained in the most consumed whole grain foods and their individual contribution to the total consumption of whole grains [13].

### Statistical analysis

The objective of the analysis was to perform a quantitative evaluation of the relationship between whole grain consumption and the occurrence of T2D, which should serve as a basis for a recommendation on the daily consumption of whole grains. We chose the percentage of subjects presenting T2D (rate of T2D) as the primary outcome for the meta-analysis. Each category of whole grain intake was considered as a specific statistical unit (named a “statistical series”). We used a hierarchical mixed least square linear regression model, combining a similar approach to the one used by Lund and Bland (2006) [14] to model the dose-response relationship between whole grain intake and occurrence of T2D, with the addition of a hierarchical model [15] to account for the hierarchical structure of the data (series nested in studies). The rate of T2D was taken as the outcome variable in a meta-regression using a random-effect approach treating each series as a cluster [16], with whole grain intake as a predictor. The series were weighted by size, using the number of subjects in the series. The *P*-value for the Wald test comparing the meta-regression slope to 0 was compared to 0.05. A *P*-value below 0.05 was considered as evidence of a significant relationship between whole grain intake and the T2D rate. The potential for departure from linearity was tested by adding a quadratic component to the univariate model. A non-significant quadratic component (*P* > 0.05) was considered as evidence of absence of departure from linearity.

The Cochran Q statistic was computed for both the intercept and the slope of the meta-regression model to quantify between-study heterogeneity. The influence of each individual study on the results was examined by repeating the analysis while omitting each study one at a time. The effects of potential covariates that could influence the outcome variable were adjusted for in the regression model as a fixed effect in a bivariate approach, with adjustments on whole grain dose and each covariate one at a time. The covariates considered were sex (% males), age (mean), country where the study was carried out, study design, mode of report of whole grain intake in the original publication (whole grain food or whole grain ingredient), and duration of follow up (for cohort studies only).

The potential for publication bias was explored by producing a Funnel plot, plotting standard error of effect versus estimate of effect-size for each study and by computing the Kendall’s rank correlation test statistic (Kendall’s tau) between the standardized effect size and the standard errors of these effects as proposed by Begg and Mazumdar [17].

SAS software version 9.2 (SAS Institute Inc., Cary NC, USA) was used for all the descriptive and meta-regression computations. R version 2.15.2 (the R Foundation for Statistical Computing, Vienna, Austria) was used for the analyses on publication bias.

## Results

### Characteristics of selected studies

Eight studies, described in six publications [18–23], were included in the meta-regression analysis (Fig 1), with a total of 15,573 cases of T2D among 316,051 participants (4.9%). Five of the studies were from the United States [19,21,22], one from Finland [20], one from Sweden [23] and one from Iran [18]. All studies but one [18] were prospective cohort studies, with follow-up durations ranging from 6 to 22 years. The remaining study had a cross-sectional design. All studies were considered by the reviewers to have been performed according to appropriate methodological standards. Table 1 summarizes the characteristics of the included studies. Four of the studies were subdivided into series by quintiles of whole grain consumption, two by quartiles, one by tertiles and one by six arbitrary cut-offs, resulting in 37 analyzed series. Overall, whole grain consumption ranged from 2.2 to 154.0 g/d of whole grain ingredients (median values of individual series). The five US studies had similar ranges from a few g/d up to 30–50 g/d, while the three other studies reached higher daily consumption levels (from a few g/d up to 117 g/d in the Iranian study, from 21 g/d up to 77 g/d in the Swedish study and from 40 g/d up to 154 g/d in the Finnish study). The rate of occurrence of T2D in the studies varied from 2.7% to 8.0%. Two of the US studies had the highest levels of occurrence of T2D (6.7% in the Health Professionals Follow-up Study, HPFS, and 8.0% in Nurses’ Health Study I, NHS I), while the other three US studies had lower T2D rates (between 2.7% and 4.8%; Table 1). The Swedish, Finnish and Iranian studies had intermediate rates (5.1%, 3.6% and 3.3%, respectively).

### Meta-regression analysis: dose-response relationship between whole grain consumption and occurrence of type 2 diabetes

**Fig 2:**
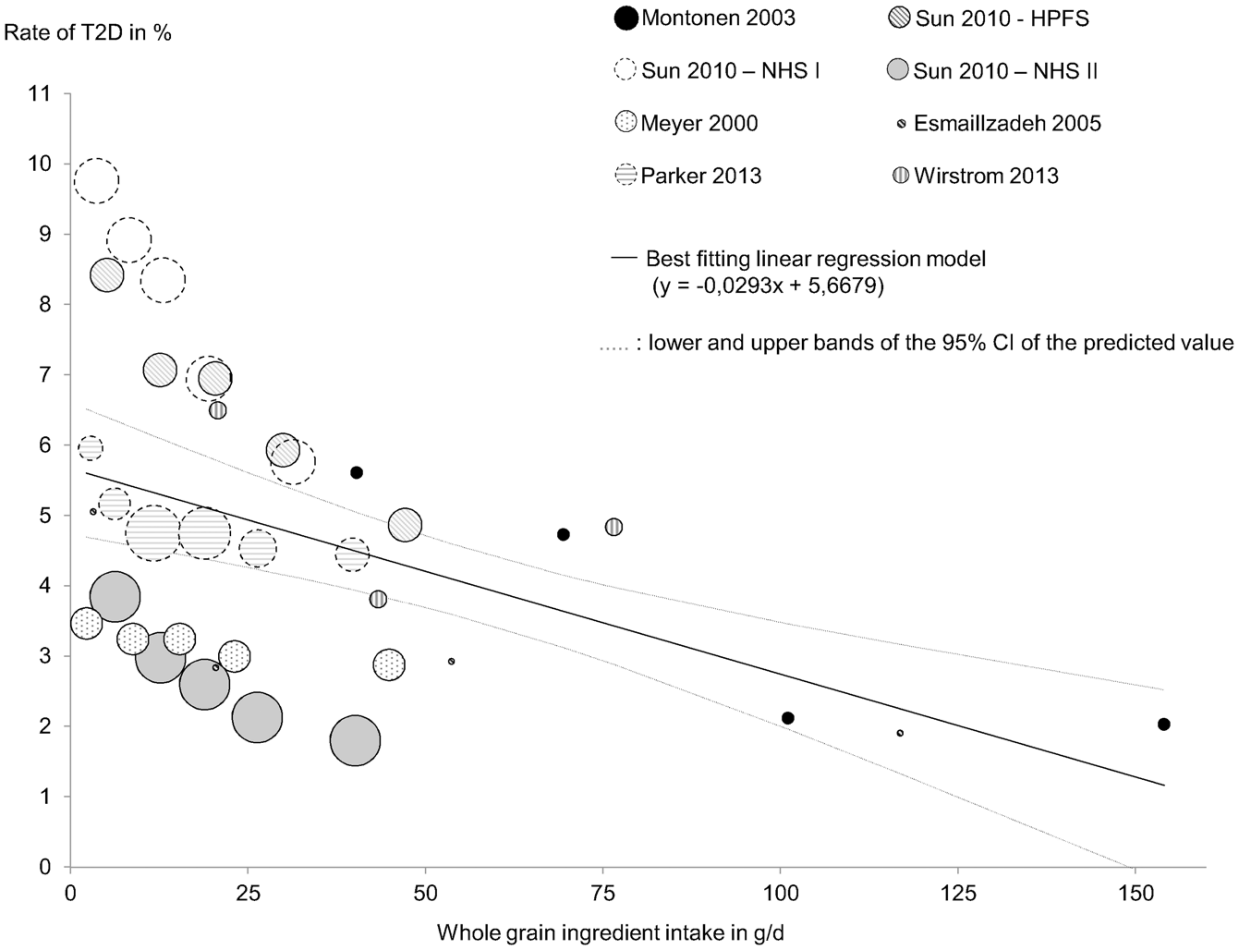
Meta-regression analysis between whole grain intake and occurrence of type 2 diabetes. The dose-response meta-regression analysis between whole grain intake and occurrence of type 2 diabetes (T2D) was performed by using a hierarchical mixed least square linear regression model, with T2D rate (% of cases) as the outcome variable and whole grain intake (in g/d of whole grain ingredients) as the predictor. Each category of whole grain intake was considered as a specific statistical unit (called a “statistical series”). Eight studies were included in the meta-regression analysis. Four of the studies were subdivided into series by quintiles of whole grain consumption, two by quartiles, one by tertiles and one by six arbitrary cut-offs, resulting in 37 analyzed series in total. The size of the circles reflects the number of subjects included in each individual series. See S2 Fig for a semi-log representation. T2D, type 2 diabetes. WG, whole grains.

There was a significant inverse association between occurrence of T2D and whole grain intakes, with a slope of -0.000293 (95% CI: -0.000424, -0.000161; *P* <0.0001), i.e., an overall reduction of 0.3% in the incidence of T2D for each additional 10 g of whole grain ingredient consumed per day (Fig 2). The quadratic component was not statistically significant in the quadratic regression model (*P* = 0.26), which indicated no evidence of departure from linearity, thus confirming that the chosen linear regression model was adequate. Bivariate meta-regression analyses were performed to assess the potential effects of age, sex, country, study design, mode of report of whole grain intakes (amounts of either whole grain ingredients or whole grain foods) and duration of follow-up (for cohort studies only). All explored covariates except age had a statistically significant influence on T2D risk (see S2 Table). However, none of these variables acted as a confounder of the effect of whole grain intake, and the relationship between whole grain intake and occurrence of T2D remained significant when adjusting on each of the covariates (Table 2). These results therefore suggest that the association between whole grain consumption and occurrence of T2D was not due to a confounding effect of any of the tested covariates.

**Table 2:** Bivariate meta-regression analyses performed on whole grain intake and occurrence of type 2 diabetes.

Name of covariate	Slope for the effect of WG on T2D rate, adjusted for individual covariate	*P* value (Wald test) for the effect of WG on T2D rate, adjusted for individual covariate
Age	-0.000281	<0.0001
Sex	-0.000372	<0.0001
Country (Iran, United States, Finland)	-0.000363	<0.0001
Study design (cohort or cross-sectional)	-0.000275	<0.0001
Mode of report of WG intake (food or ingredient)	-0.000242	<0.0001
Effect of follow-up duration^1^	-0.000355	<0.0001

^1^ Analyzed on the seven cohort studies only.

The dose-response meta-regression analysis between whole grain intake and occurrence of type 2 diabetes (T2D) was performed by using a hierarchical mixed least square linear regression model, with T2D rate as the outcome variable and whole grain intake as the predictor. The *P*-value for the Wald test comparing the meta-regression slope to 0 was compared to 0.05. A *P*-value below 0.05 was considered as evidence of a significant relationship between whole grain intake and the T2D rate. The effects of potential covariates that could influence the outcome variable were adjusted for as a fixed effect in a bivariate regression model, with adjustments on whole grain dose and each covariate one at a time. The covariates considered were sex (% males), age (mean), country where the study was carried out, study design, mode of report of whole grain intake in the original publication (whole grain food or whole grain ingredient), and duration of follow up (for cohort studies only). T2D, type 2 diabetes. WG, whole grains.

### Exploration of heterogeneity, influence of individual studies and publication bias

The Cochran Q test was not significant for the regression slope (Q = 0.11, *P* = 0.999), indicating that there was no evidence of heterogeneity between studies in the relationship between whole grain intake and T2D occurrence. However, the Cochran Q test was significant for the intercept (Q = 218.1, *P* < 0.0001), indicating that there was a significant heterogeneity in the T2D rates across studies, which was to be expected given the observed variability in the rate of T2D in individual studies (Table 1).

Repeating the meta-regression analysis by omitting each study one at a time showed that whatever the excluded study, the relationship between whole grain intake and occurrence of T2D remained statistically significant (see S3 Table). There was no publication bias on the basis of visual inspection of the funnel plot, with no apparent evidence of asymmetry (see S3 Fig), or on the basis of the non-significant Kendall’s Tau statistic (Kendall’s Tau = -0.1429, *P* = 0.7), although the number of studies was low.

## Discussion

Our meta-analysis showed a statistically significant inverse association between intake of whole grains and occurrence of T2D, which was independent of sex, age, country, study design, duration of follow up, and mode of report of whole grain intakes (whole grain food or ingredient). This observation confirms the findings of previous systematic reviews [24] and meta-analyses [1,2], which included a slightly different set of studies, used different statistical methodologies and did not provide full quantitative information. More importantly, our meta-regression model makes it possible to estimate the decrease in risk of T2D corresponding to various changes in whole grain intakes. For example, an increase of 20 g/d in the intake of whole grain ingredients would result in an absolute reduction of 0.6% in the risk of T2D; considering a 4.9% risk of T2D in the whole population, this corresponds to a 12% relative reduction in the risk of T2D. Our statistical analysis indicates that the relationship can be assumed to be linear. However, as can be seen in Fig 2, most of the analyzed data supports linearity up to an intake of whole grain ingredients of approximately 50 g/d, data with higher levels of consumption being scarce. This model could be translated by saying that a population consuming 45 g/d of whole grain ingredients would decrease its risk of T2D by 20% as compared to consuming 7.5 g/d of whole grain ingredients.

This would correspond to the situation in several Western countries, such as in the United States, where the current average intake of whole grain ingredients is approximately 10.5 g/d [25], or in the United Kingdom, where the median intake is approximately 14 g/d [26]. Hence, increasing the daily intake up to 45 g/d, which corresponds approximately to three servings of whole grain foods (assuming one serving of whole grain foods equals approximately 15 g of whole grain ingredients), would provide a significant health benefit in such populations by inducing a relative reduction in the risk of T2D of approximately 18%. In the Nordic countries, where the current intake of whole grains is much higher, another simulation from our meta-regression model suggests that a 21% relative reduction in the risk of T2D can be reached when the daily intake in whole grain ingredients increases from the current average intake of 46 g/d [27,28] to 76 g/d (i.e., an increase of approximately two servings of whole grain foods).

As a comparison, a recent meta-analysis of prospective cohorts [29] showed that increasing fiber intake from 15 g/d, which is approximately the average intake in the United States [30], to the recommended intake of 25g/d [31,32] would lower the relative risk of T2D by 9%. Diabetes (type 1 or T2D) affects 8.3% of the US population, i.e., 25.8 million people [33], and 347 million people worldwide [34]. A relative decrease of the T2D risk by more than 10% can thus be considered as a highly relevant prevention rate.

Among the plausible mechanisms that can trigger this beneficial impact on T2D, the hypothesis that whole grains may favor a better control of glucose and insulin metabolisms is supported by several experimental human studies [9] and by a meta-analysis of 14 prospective studies which concluded that a higher intake of whole grain foods was associated with lower fasting glucose and insulin concentrations [35]. The effect of whole grains could be explained in part by their content in several nutrients and phytochemicals such as fibers, minerals, vitamins, phenolic compounds, lignans or phytic acid [9,36].

Our study had several limitations that may affect the interpretation of the results. Firstly, there was a large range of whole grain intakes across studies: in three studies [18,20,23], corresponding to only 1.7% of the total number of subjects included in the meta-analysis, intake of whole grain ingredients was much higher (> 50 g/d) than in the five remaining US studies [19,21,22]. Therefore, despite being consistent with results obtained for smaller intakes, results for high whole grain intakes should be considered more cautiously. Secondly, and in addition to potential inadequate reporting of the intake of whole grain foods by subjects, as it may occur for any other food, the methodology used to assess whole grain content of these foods varied among the included studies. While four cohorts (accounting for 64% of the total number of subjects included in the meta-analysis) directly provided actual intakes of whole grain ingredients [21,23], the other studies [18–20,22] reported intakes of whole grain foods. As detailed in the material and method section, we extrapolated the whole grain ingredient content assuming that whole grain foods contained 51% of whole grain ingredients on average. This assumption resulted in approximations which are likely to have led to cautious conclusions. Indeed, whole grain intakes are underestimated when using the 25% threshold proposed by Jacobs *et al*. (1998) [10] rather than the 51% threshold, therefore leading to an overestimation of the effect of whole grains on the risk of disease. Importantly, the mode of reporting whole grain intakes (foods or ingredients) in original publications was considered as a covariate in the meta-regression analysis, and this did not significantly affect the results. Thirdly, the observed association between whole grain intake and the risk of T2D may result from unmeasured or residual confounding by other dietary or lifestyle factors, such as a higher level of physical activity, a lower prevalence of smoking, overweight or obesity. Since these factors were not considered in the meta-regression analysis, the strength of the inverse association between whole grain intake and the risk of T2D may have been overestimated, although probably not to a large extent. Fourthly, most of the population included in the meta-analysis was living in the United States, which may draw the generalizability of our findings into question. Still, they appear relevant for other Western countries with similar overall dietary patterns and similar risk of T2D.

Our study also had several strengths. Firstly, this was the first meta-analysis evaluating the quantitative relationship between whole grain intakes and the risk of T2D by using a meta-regression model of T2D occurrence rates on whole grain intakes. Unlike classical meta-analyses which usually focus only on the lowest and highest categories of dietary intakes, the whole information on whole grain intakes was used, leading to a more accurate estimate of the quantitative association between whole grain intakes and the risk of T2D. Furthermore, because of the high degree of heterogeneity between studies in the ranges of whole grain intakes, similar levels of whole grain consumption can be the lowest category for some studies and the highest for others. It can therefore not be assumed that the highest and lowest categories of whole grain intakes in all studies have the same risk of T2D, a point that has been overlooked by all meta-analyses focusing on the relative risks between highest and lowest categories of intake. This observation further justifies the choice of our statistical methodology. Secondly, although our meta-analysis included only eight studies, this represented more than 15,500 cases of T2D, for a total of 316,051 participants. Thirdly, most of the included studies had a prospective design, reducing the risk that our findings could be explained by recall or selection bias. Fourthly, the meta-analysis displayed several indicators of robustness, including consistent findings across studies with no evidence of between-study heterogeneity on the regression slope, no strong influence of one single study on the overall result, no confounding effect of any of the tested covariates, no evidence of publication bias and no evidence of departure from linearity. The latter confirmed that the linear model was appropriate.

Results from our quantitative meta-analysis suggest that any increase in whole grain consumption would be beneficial toward the prevention of T2D. The dose-response relationship is a quantitative tool which can contribute to the setting up of recommendations for the daily intake of whole grains, by considering the benefits of an increased intake. For instance, setting a recommendation for intake of whole grain ingredients at 45 g/d (approximately 3 servings of whole grain foods) in a population consuming only half a serving of whole grain foods would induce a 20% relative reduction in the risk of T2D. These results should also contribute to the adapting of recommendations to specific regions, by taking the current whole grain intake of the population into account. Helpful and realistic advice could thus be provided.

Such recommendations should be complemented with data on the quantitative relationship between whole grain intakes and other health outcomes, such as the risk of cardiovascular diseases, obesity or overall mortality, for which similar analyses are ongoing. Finally, in setting up a quantitative recommendation for whole grain consumption, stakeholders should also provide consumers with clear labeling of whole grain foods and, more generally, with effective means of following recommendations.

## Supporting Information

S1 Checklist MOOSE checklist for the Reporting of Meta-analyses of Observational Studies. (XLSX)

S1 Fig Sets of key words used in the literature search. • stands for “OR”. LDL, Low-density lipoprotein.
(PPTX)

S2 Fig Semi-log representation of the meta-regression analysis between logarithm of whole grain intake (in g/d) and occurrence of type 2 diabetes (% of cases). (PPTX)

S3 Fig Exploration of publication bias (Funnel plot). The potential for publication bias was explored by producing a Funnel plot, plotting standard error of effect versus estimate of effect-size for each study and by computing the Kendall’s rank correlation test statistic (Kendall’s tau) between the standardized effect size and the standard errors of these effects.
(PPTX)

S1 Protocol Protocol of the systematic review as published on the PROSPERO register. (PDF)

S1 Table List of the 210 articles selected for full text evaluation. The outcome of the selection process is indicated for each article (with justification for exclusion).
(XLSX)

S2 Table Bivariate meta-regression analyses: effect of individual covariates on the occurrence of type 2 diabetes. The dose-response meta-regression analysis between whole grain intake and occurrence of type 2 diabetes (T2D) was performed by using a hierarchical mixed least square linear regression model, with T2D rate as the outcome variable and whole grain intake as the predictor. The effects of potential covariates that could influence the outcome variable were adjusted for as a fixed effect in a bivariate regression model, with adjustments on whole grain dose and each covariate one at a time. The covariates considered were sex (% males), age (mean), country where the study was carried out, study design, mode of report of whole grain intake in the original publication (whole grain food or whole grain ingredient), and duration of follow up (for cohort studies only). The P-value for the Wald test comparing the meta-regression slope to 0 was compared to 0.05. A P-value below 0.05 was considered as evidence of a significant relationship between the considered covariate and the T2D rate. T2D, type 2 diabetes. WG, whole grains.
(DOCX)

S3 Table Influence of individual studies on the results of the meta- regression analysis between whole grain intake and occurrence of type 2 diabetes. The influence of each individual study on the results was examined by repeating the meta-regression analysis while omitting each study one at a time.
(DOCX)
