# Supplementary material for: Systematic Review and Meta-Analysis of Human Studies to Support a Quantitative Recommendation for Whole Grain Intake in Relation to Type 2 Diabetes
Source: PLoS One. 2015 Jun 22;10(6):e0131377. doi: 10.1371/journal.pone.0131377 (PMC4476805; doi:10.1371/journal.pone.0131377)
Supplement: S1 Fig — • stands for “OR”. LDL, Low-density lipoprotein. (PPTX) [file pone.0131377.s002.pptx]

## Slide 1
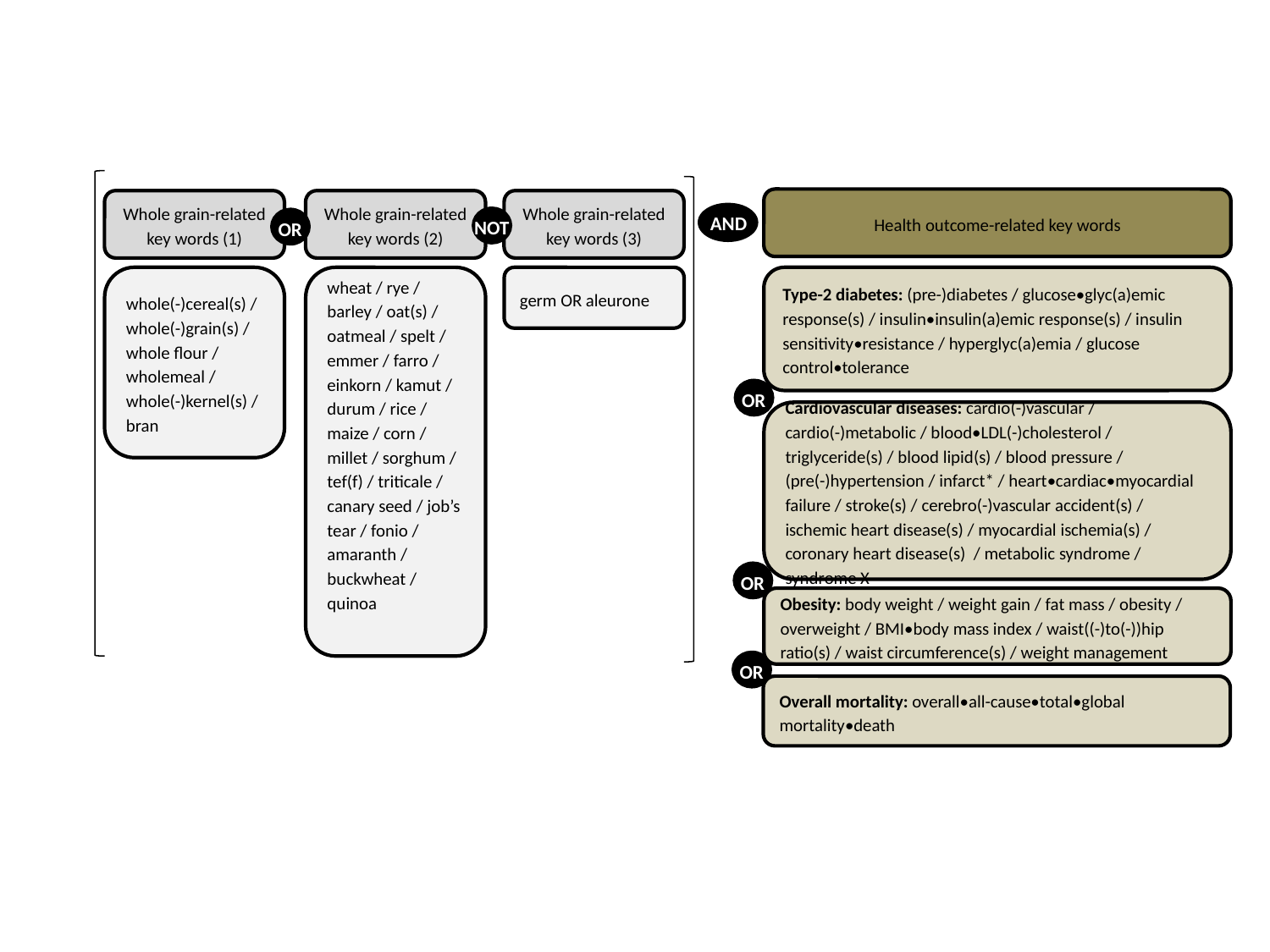

Health outcome-related key words
Whole grain-related key words (2)
Whole grain-related key words (1)
Whole grain-related key words (3)
AND
NOT
OR
wheat / rye / barley / oat(s) / oatmeal / spelt / emmer / farro / einkorn / kamut / durum / rice / maize / corn / millet / sorghum / tef(f) / triticale / canary seed / job’s tear / fonio / amaranth / buckwheat / quinoa
whole(-)cereal(s) / whole(-)grain(s) / whole flour / wholemeal / whole(-)kernel(s) / bran
germ OR aleurone
Type-2 diabetes: (pre-)diabetes / glucose•glyc(a)emic response(s) / insulin•insulin(a)emic response(s) / insulin sensitivity•resistance / hyperglyc(a)emia / glucose control•tolerance
OR
Cardiovascular diseases: cardio(-)vascular / cardio(-)metabolic / blood•LDL(-)cholesterol / triglyceride(s) / blood lipid(s) / blood pressure / (pre(-)hypertension / infarct* / heart•cardiac•myocardial failure / stroke(s) / cerebro(-)vascular accident(s) / ischemic heart disease(s) / myocardial ischemia(s) / coronary heart disease(s) / metabolic syndrome / syndrome X
OR
Obesity: body weight / weight gain / fat mass / obesity / overweight / BMI•body mass index / waist((-)to(-))hip ratio(s) / waist circumference(s) / weight management
OR
Overall mortality: overall•all-cause•total•global mortality•death
