# Supplementary material for: Systematic Review and Meta-Analysis of Human Studies to Support a Quantitative Recommendation for Whole Grain Intake in Relation to Type 2 Diabetes
Source: PLoS One. 2015 Jun 22;10(6):e0131377. doi: 10.1371/journal.pone.0131377 (PMC4476805; doi:10.1371/journal.pone.0131377)
Supplement: S2 Fig — (PPTX) [file pone.0131377.s003.pptx]

## Slide 1
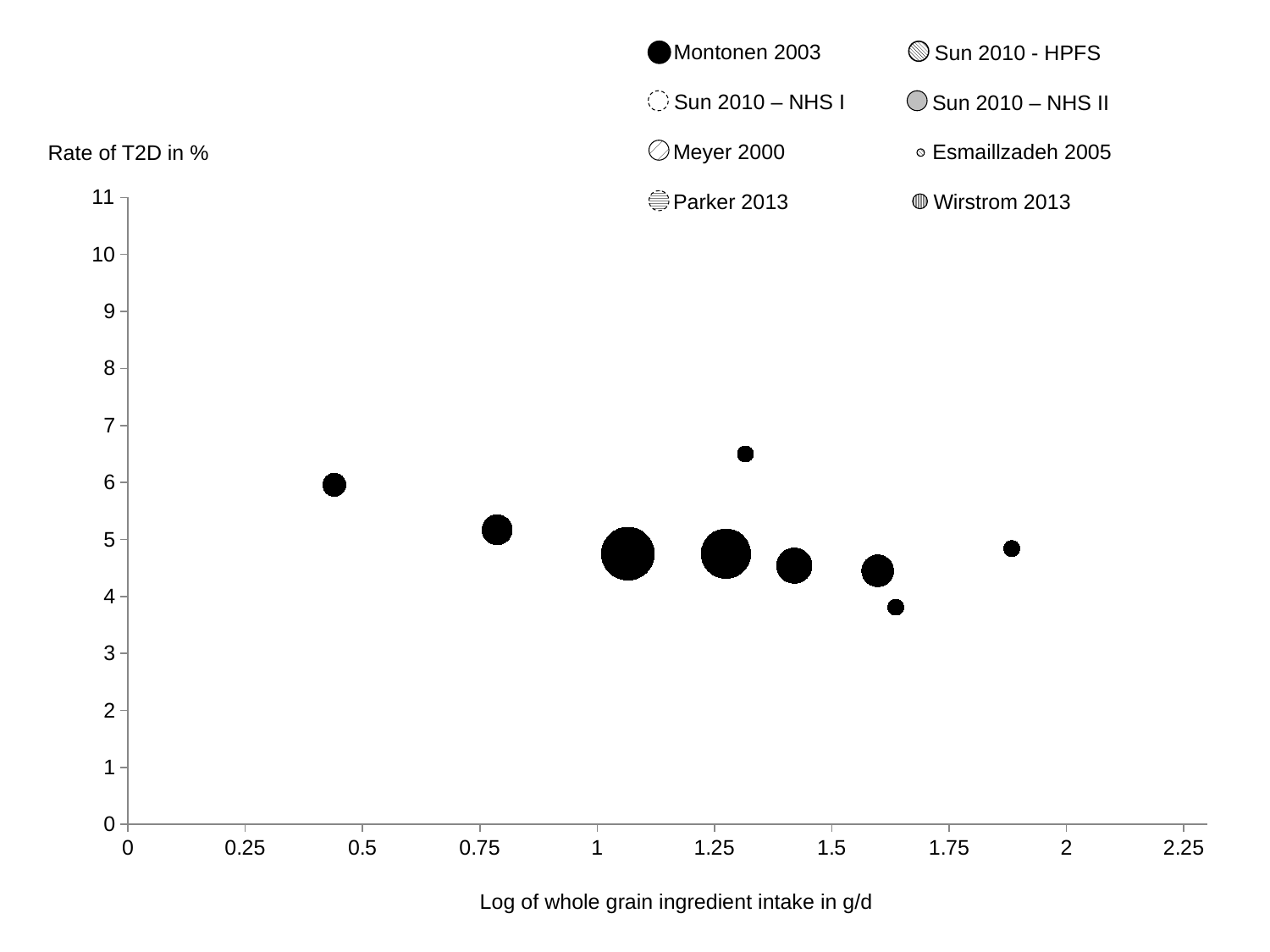

Montonen 2003
Sun 2010 - HPFS
Sun 2010 – NHS I
Sun 2010 – NHS II
### Chart
| Category | | | | | | | | | | | |
|---|---|---|---|---|---|---|---|---|---|---|---|Meyer 2000
Esmaillzadeh 2005
Rate of T2D in %
Parker 2013
Wirstrom 2013
Log of whole grain ingredient intake in g/d
