# Supplementary figures and images for: Systematic Review and Meta-Analysis of Human Studies to Support a Quantitative Recommendation for Whole Grain Intake in Relation to Type 2 Diabetes
Source: PLoS One. 2015 Jun 22;10(6):e0131377. doi: 10.1371/journal.pone.0131377 (PMC4476805; doi:10.1371/journal.pone.0131377)

## Slide 1
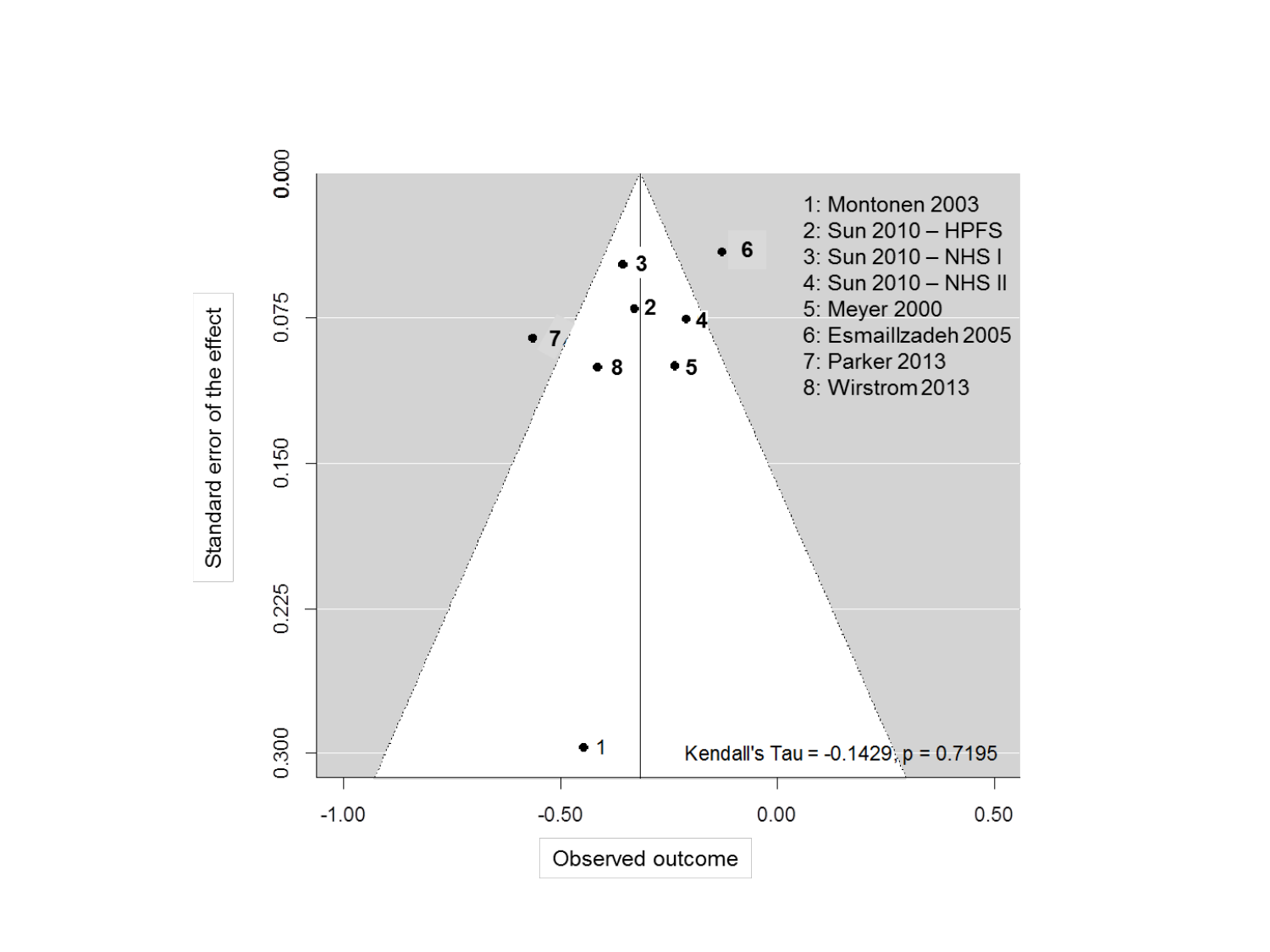

Supplement: S3 Fig — The potential for publication bias was explored by producing a Funnel plot, plotting standard error of effect versus estimate of effect-size for each study and by computing the Kendall’s rank correlation test statistic (Kendall’s tau) between the standardized effect size and the standard errors of these effects. (PPTX) [file pone.0131377.s004.pptx]
