# Supplementary material for: Systematic Review and Meta-Analysis of Human Studies to Support a Quantitative Recommendation for Whole Grain Intake in Relation to Type 2 Diabetes
Source: PLoS One. 2015 Jun 22;10(6):e0131377. doi: 10.1371/journal.pone.0131377 (PMC4476805; doi:10.1371/journal.pone.0131377)
Supplement: S1 Protocol — (PDF) [file pone.0131377.s005.pdf]

PROSPERO International prospective register of systematic reviews

Health benefits of wholegrain: a systematic review of clinical studies to propose a quantitative recommendation for a daily intake

Aurelie Chanson-Rolle, Jenni Lappi, Alexandra Meynier, Sophie Vinoy, Véronique Braesco, Kaisa Poutanen

Citation

Aurelie Chanson-Rolle, Jenni Lappi, Alexandra Meynier, Sophie Vinoy, Véronique Braesco, Kaisa Poutanen. Health benefits of wholegrain: a systematic review of clinical studies to propose a quantitative recommendation for a daily intake. PROSPERO 2013:CRD42013006925 Available from
http://www.crd.york.ac.uk/PROSPERO_REBRANDING/display_record.asp?ID=CRD42013006925

Review question(s)

Is there sufficient evidence from published human studies to derive a quantitative recommendation for the daily consumption of wholegrain in relation to various favorable health outcomes?

Searches

The following bibliographic databases had been searched: MEDLINE (via PubMed), Cochrane Central Register of Controlled Trials (via the Cochrane library), Cochrane Database of Systematic Reviews (via the Cochrane library), all from 01/01/1993 to 31/12/2012.

The searches had been limited to human studies and to studies published in English (for Medline only), but without any limit on publication type.

The following keywords were searched in Title/abstract for Medline and in Title/abstract/keywords for the Cochrane Library: ((“whole cereal” OR “whole-cereal” OR “whole cereals” OR “whole-cereals” OR “whole grain” OR “whole-grain” OR “whole grains” OR “whole-grains” OR “wholegrain” OR “wholegrains” OR “whole flour” OR “wholemeal” OR “whole kernel” OR “whole-kernel” OR “whole kernels” OR “whole-kernels” OR bran

OR barley OR oat OR oatmeal OR oats OR spelt OR emmer OR farro OR einkorn OR kamut OR durum OR rice OR maize OR corn OR millet OR sorghum OR teff OR tef OR triticale OR “canary seed” OR “job’s tear” OR fonio OR amaranth OR buckwheat OR quinoa OR wheat OR rye) NOT (germ OR aleurone))

AND (cardiovascular OR “cardio-vascular” OR cardiometabolic OR “cardio-metabolic” OR “blood cholesterol” OR “LDL cholesterol” OR “LDL-cholesterol” OR triglyceride OR triglycerides OR “blood lipid” OR “blood lipids” OR “blood pressure” OR hypertension OR prehypertension OR “pre-hypertension” OR infarct* OR “heart failure” OR “cardiac failure” OR “myocardial failure” OR stroke OR strokes OR “cerebrovascular accident” OR “cerebrovascular accidents” OR “cerebro-vascular accident” OR “cerebro-vascular accidents” OR “ischemic heart disease” OR “ischemic heart diseases” OR “myocardial ischemia” OR “myocardial ischemias” OR “coronary heart disease” OR “coronary heart diseases” OR “metabolic syndrome” OR “syndrome X” OR “X syndrome” OR diabetes OR prediabetes OR “pre-diabetes” OR “glucose response” OR “glycemic response” OR “glycaemic response” OR “insulin response” OR “insulinemic response” OR “insulinaemic response” OR “glucose responses” OR “glycemic responses” OR “glycaemic responses” OR “insulin responses” OR “insulinemic responses” OR “insulinaemic responses” OR “insulin sensitivity” OR “insulin resistance” OR hyperglycemia OR hyperglycaemia OR “glucose control” OR “glucose tolerance” OR “body weight” OR “weight gain” OR “fat mass” OR obesity OR overweight OR BMI OR “body mass index” OR “waist to hip ratio” OR “waist-to-hip ratio” OR “waist hip ratio” OR “waist-hip ratio” OR “waist/hip ratio” OR “waist to hip ratios” OR “waist-to-hip ratios” OR “waist hip ratios” OR “waist-hip ratios” OR “waist/hip ratios” OR “waist circumference” OR “waist circumferences” OR “weight management” OR “overall mortality” OR “all-cause mortality” OR “total mortality” OR “global mortality” OR “overall death” OR “all-cause death” OR “total death” OR “global death”).

In addition, the reference lists of included studies and reviews were searched in order to find other potentially eligible

studies.

Paper screening and selection were performed independently by 3 team members (ACR, AM, JL) and discrepancies were resolved by discussions at each stage. All retrieved studies were screened by the 3 collaborators on the basis of the reading of titles and abstracts to select studies to be assessed further, and all potentially relevant studies were further considered by reading the full texts.

UPDATE OF THE LITERATURE SEARCH: An update of the literature search will be performed to search for relevant papers published between 01/01/2013 and March 2015. The same set of key words and the same strategy for paper screening and selection as described above will be used, except that paper screening and selection will be performed by two team members (ACR and AM).

Types of study to be included

Observational (including cross-sectional, case-control and cohort studies) and intervention studies addressing the relationship between wholegrain consumption and one of the health outcomes of interest (overall mortality, obesity, cardiovascular diseases, type-2 diabetes and associated risk factors), and providing quantitative information on daily intake of wholegrain

Condition or domain being studied

It has been proposed that wholegrain consumption may have a beneficial impact on several health outcomes including overall mortality, obesity, cardiovascular diseases, type-2 diabetes and associated risk factors. Those outcomes should be considered in the attempt of deriving a quantitative recommendation for daily wholegrain intake.

Participants/ population

Inclusion: overall human population except children younger than 2 years old.

Exclusion: studies performed in patients suffering from cancer, renal insufficiency, type 1 diabetes or other pathologies than cardiovascular diseases or type 2 diabetes, studies performed in hypercholesterolemic / hypertriglyceridemic / hypertensive/ diabetic subjects when treated with hypolipemic / hypotensive / hypoglycemic drugs, studies performed in hospitalized or long-term institutionalized patients or in undernourished populations.

Intervention(s), exposure(s)

Observational studies: categories of wholegrain intakes.

Intervention studies: consumption of wholegrain foods or of diets high in wholegrain foods.

Comparator(s)/ control

Observational studies: lowest categories of wholegrain intakes.

Intervention studies: consumption of refined-grain foods or of diets high in refined-grain foods or of usual diet.

Context

Excluded studies: studies with missing information (e.g., no quantitative information on daily intake of wholegrain), for which no appropriate answer is received from the authors and for which the missing information cannot be calculated from the publication; redundant studies (when several publications are available from the same study, the publications with the lowest duration of follow up are excluded); studies evaluating acute or a short term effect of wholegrain intake.

Outcome(s)

Primary outcomes

Occurrence of cardiovascular diseases, type-2 diabetes, overweight, obesity, metabolic syndrome, overall mortality, mortality related to cardiovascular diseases

Secondary outcomes

Changes in body weight, body mass index, lean body mass, body fat, central/abdominal fat, blood pressure, fasting blood concentration of lipids (LDL cholesterol, total cholesterol, HDL-cholesterol & triglycerides), glucose tolerance

(oral glucose tolerance test, if measured after continuous/chronic consumption of wholegrain), glycated hemoglobin, fasting blood glucose, insulin sensitivity, first-phase insulin secretion, endothelium-dependent vasodilation (FMD), platelet aggregation, fasting homocystein blood concentration, inflammatory markers, plasminogen activator inhibitor-1 (PAI-1), arterial stiffness, recognized markers of lipid peroxidation (e.g., urine F2a isoprostanes), postprandial glycaemic and insulinaemic responses (if measured after continuous/chronic consumption of wholegrain), fructosamine, fasting adiponectin blood concentration, fasting C peptide blood concentration.

Data extraction, (selection and coding)

For studies that fulfill the inclusion criteria, three review team members (AM, VB and ACR) will independently extract relevant information from the full texts of the selected studies, using a standardised, pre-piloted form. This form will be used for assessment of study quality and evidence synthesis. Extracted information:

I. General information : authors, article title, year of publication and references.

II. Study characteristics: design, country, randomization, details of the intervention and control conditions, method used for assessment of wholegrain intake, mode of report of wholegrain intake (food or ingredient), level of consumption of wholegrain intake (quantitative information in g or servings per day, week…), background diet, duration of follow-up.

III. Participants: number of subjects in total and in each group, gender, age, baseline characteristics of the subjects.

IV. Outcomes: assessed outcomes, methods and times of measurement, methods used for statistical analyses, information for assessment of the risk of bias.

Delicate issues will be resolved through discussions between the three team members. Missing data will be requested from study authors, if required.

UPDATE OF THE LITERATURE SEARCH: Relevant information will be extracted from the additional relevant studies that will be identified through the updated literature search (01/01/2013 - March 2015) following the same methodology as described above, except that data extraction will be performed by two team members (ACR and AM).

Risk of bias (quality) assessment

The three investigators involved in data extraction will assess the risk of bias in included studies by using a customized tool with different domains depending on the study design. For observational studies, the main domains to be evaluated will be: study design (cohort, case-control or cross-sectional study), selection of participants (report of subject inclusion and exclusion criteria), report of baseline characteristics of subjects for relevant parameters, mode of report of wholegrain intake, statistical methods, adjustment for confounding variables, declaration of conflict of interest or identification of funding. For intervention studies, the main domains to be evaluated will be: randomization, blinding, sample size & power calculation, selection of participants (report of subject inclusion and exclusion criteria), report of baseline characteristics of subjects for relevant parameters, information on background dietary habits, record of dietary intakes during the study, mode of report of wholegrain intake, report of the compliance of subjects with intervention, comparison between study groups at baseline, statistical methods, adjustment for confounding variables, declaration of conflict of interest or identification of funding.

Each of these key components of methodological quality will be assessed on a Yes/No (or unknown)/Partially basis.

Strategy for data synthesis

We aim to provide a narrative synthesis of the findings from the included studies, structured around the type of health outcome and type of study. In order to reach the objective of the systematic review, we plan to perform quantitative meta-analyses for primary outcomes for which the included studies will be sufficiently homogenous. The aim of these meta-analyses (one meta-analysis per outcome) will be to quantify the relationship between wholegrain consumption and the selected health outcome and, if possible, to identify a relevant threshold of consumption. These quantitative syntheses will be conducted on aggregate data. Classical, frequentist approaches for meta-analysis and meta-regression will be used, and random effects will be preferred to fixed effect models. Effect of potential covariates (eg sex, age, country, study design, study duration) will be considered.

Heterogeneity will be assessed by computing the usual tau^2, I^2, H^2 and Cochran Q statistic.

Influence of each individual study on the results will be studied by repeating the analysis while omitting each study at a time. We will also assess evidence of publication bias by the mean of funnel plots and asymmetry tests (Kendall's tau).

Analysis of subgroups or subsets
If relevant and if the necessary data are available, we will conduct exploratory analyses to study the influence of gender, age, follow-up duration, study design (eg for observational studies: cross-sectional/case-control study vs cohort study), country of the study, mode of report of wholegrain intake [food or ingredient]. Additional exploratory analyses may be performed if considered as of interest. These exploratory analyses will use meta-regression techniques and/or subgroup analyses.

Dissemination plans
Conferences/posters; papers to be submitted to peer-reviewed journals in the relevant fields.

Contact details for further information
Aurelie Chanson-Rolle

aurelie.chanson-rolle@vab-nutrition.com

Organisational affiliation of the review
None

Review team
Dr Aurelie Chanson-Rolle, VAB-nutrition (France)
Mrs Jenni Lappi, University of Eastern Finland
Dr Alexandra Meynier, Mondelez France R&D
Dr Sophie Vinoy, Mondelez France R&D
Dr Véronique Braesco, VAB-nutrition (France)
Dr Kaisa Poutanen, VTT Technical Research Centre of Finland

Collaborators
Dr François Aubin, Cardinal Systems (France)

Anticipated or actual start date
01 March 2013

Anticipated completion date
30 June 2016

Funding sources/sponsors
The review is sponsored by MONDELEZ FRANCE R&D SAS (6 rue René Razel – Bâtiment K. 91400 SACLAY. FRANCE)

Conflicts of interest
Alexandra Meynier and Sophie Vinoy are employees of Mondelez FRANCE R&D SAS.

Aurelie Chanson-Rolle and Veronique Braesco have received fees from Mondelez FRANCE R&D SAS for performing the systematic review.

The other team members declare that they have no known conflicts of interest (Kaisa Poutanen is employee of VTT and Jenni Lappi is employee of University of Eastern Finland).

Language

English

Country
Finland, France

Subject index terms status
Subject indexing assigned by CRD

Subject index terms
Bread; Cereals; Humans; Nutritive Value

Stage of review
Ongoing

Date of registration in PROSPERO
20 December 2013

Date of publication of this revision
11 March 2015

Stage of review at time of this submission	Started	Completed
Preliminary searches	Yes	Yes
Piloting of the study selection process	Yes	Yes
Formal screening of search results against eligibility criteria	Yes	Yes
Data extraction	Yes	No
Risk of bias (quality) assessment	Yes	No
Data analysis	Yes	No

PROSPERO
International prospective register of systematic reviews
The information in this record has been provided by the named contact for this review. CRD has accepted this information in good faith and registered the review in PROSPERO. CRD bears no responsibility or liability for the content of this registration record, any associated files or external websites.
