# Supplementary material for: Systematic Review and Meta-Analysis of Human Studies to Support a Quantitative Recommendation for Whole Grain Intake in Relation to Type 2 Diabetes
Source: PLoS One. 2015 Jun 22;10(6):e0131377. doi: 10.1371/journal.pone.0131377 (PMC4476805; doi:10.1371/journal.pone.0131377)
Supplement: S2 Table — The dose-response meta-regression analysis between whole grain intake and occurrence of type 2 diabetes (T2D) was performed by using a hierarchical mixed least square linear regression model, with T2D rate as the outcome variable and whole grain intake as the predictor. The effects of potential covariates that could influence the outcome variable were adjusted for as a fixed effect in a bivariate regression model, with adjustments on whole grain dose and each covariate one at a time. The covariates considered were sex (% males), age (mean), country where the study was carried out, study design, mode of report of whole grain intake in the original publication (whole grain food or whole grain ingredient), and duration of follow up (for cohort studies only). The P-value for the Wald test comparing the meta-regression slope to 0 was compared to 0.05. A P-value below 0.05 was considered as evidence of a significant relationship between the considered covariate and the T2D rate. T2D, type 2 diabetes. WG, whole grains. (DOCX) [file pone.0131377.s007.docx]

| **Name of covariate** | **Covariate category (when relevant)** | **Slope for the effect of individual covariate on T2D rate** | ***P* value (Wald test) for the effect of individual covariate on T2D rate** |
| --- | --- | --- | --- |
| Effect of age |  | 0.000292 | 0.31 |
| Effect of sex |  | 0.000219 | <0.0001 |
| Effect of country | Iran | -0.019352 | 0.0047 |
|  | Sweden | -0.001799 | 0.8108 |
|  | USA | -0.011734 | 0.0738 |
|  | Finland | (ref) | (ref) |
| Effect of study design | Cohort | 0.011793 | 0.0089 |
|  | Cross-sectional | (ref) | (ref) |
| Effect of the mode of report of WG intake | Food | -0.014110 | 0.0120 |
|  | Ingredient | (ref) | (ref) |
| Effect of follow-up duration^1^ |  | 0.002083 | <0.0001 |

^1^ Analyzed on the seven cohort studies only.
