# Supplementary material for: Systematic Review and Meta-Analysis of Human Studies to Support a Quantitative Recommendation for Whole Grain Intake in Relation to Type 2 Diabetes
Source: PLoS One. 2015 Jun 22;10(6):e0131377. doi: 10.1371/journal.pone.0131377 (PMC4476805; doi:10.1371/journal.pone.0131377)
Supplement: S3 Table — The influence of each individual study on the results was examined by repeating the meta-regression analysis while omitting each study one at a time. (DOCX) [file pone.0131377.s008.docx]

| Description | Description | Estimate | 95% CI | Statistical test | p-value | Variance explained |
| --- | --- | --- | --- | --- | --- | --- |
| All eight studies (N=37) | Fit statistics | -2LL=-189.8 / AIC=-183.8/BIC=-178.9 |  |  |  |  |
|  | Between study variance (Heterogeneity) | 0.000334 | [0.000220, 0.000570] |  |  | 20.61% |
|  | Intercept | 0.056679 | [0.047180, 0.066178] | Wald: 12.113 | <.0001 |  |
|  | **Whole grain** | **-0.000293** | **[-0.000424, -0.000161]** | **Wald: -4.372** | **<.0001** |  |
| Excluding Montonen 2003  (N=33) | Fit statistics | -2LL=-166.9 / AIC=-160.9/BIC=-156.4 |  |  |  |  |
|  | Between study variance (Heterogeneity) | 0.000361 | [0.000232, 0.000637] |  |  | 15.15% |
|  | Intercept | 0.057884 | [0.046991, 0.068776] | Wald: 10.838 | <.0001 |  |
|  | **Whole grain** | **-0.000371** | **[-0.000599, -0.000143]** | **Wald: -3.191** | **0.0015** |  |
| Excluding Sun 2010 - HPFS (N=32) | Fit statistics | -2LL=-164.7 / AIC=-158.7/BIC=-154.3 |  |  |  |  |
|  | Between study variance (Heterogeneity) | 0.000328 | [0.000209, 0.000585] |  |  | 18.28% |
|  | Intercept | 0.052767 | [0.042564, 0.062970] | Wald: 10.562 | <.0001 |  |
|  | **Whole grain** | **-0.000256** | **[-0.000386, -0.000125]** | **Wald: -3.833** | **0.0001** |  |
| Excluding Sun 2010 – NHS I (N=32) | Fit statistics | -2LL=-176.8 / AIC=-170.8/BIC=-166.4 |  |  |  |  |
|  | Between study variance (Heterogeneity) | 0.000218 | [0.000138, 0.000396] |  |  | 16.72% |
|  | Intercept | 0.049118 | [0.040982, 0.057255] | Wald: 12.329 | <.0001 |  |
|  | **Whole grain** | **-0.000204** | **[-0.000307, -0.000100]** | **Wald: -3.853** | **0.0001** |  |
| Excluding Sun 2010 – NHSII (N=32) | Fit statistics | -2LL=-169.8 / AIC=-163.8/BIC=-159.4 |  |  |  |  |
|  | Between study variance (Heterogeneity) | 0.000273 | [0.000173, 0.000493] |  |  | 31.43% |
|  | Intercept | 0.061947 | [0.052144, 0.071750] | Wald: 12.906 | <.0001 |  |
|  | **Whole grain** | **-0.000330** | **[-0.000463, -0.000196]** | **Wald: -4.840** | **<.0001** |  |
| Excluding Meyer 2000 (N=32) | Fit statistics | -2LL=-165.9 / AIC=-159.9/BIC=-155.5 |  |  |  |  |
|  | Between study variance (Heterogeneity) | 0.000313 | [0.000199, 0.000561] |  |  | 29.21% |
|  | Intercept | 0.061445 | [0.051372, 0.071518] | Wald: 12.457 | <.0001 |  |
|  | **Whole grain** | **-0.000338** | **[-0.000475, -0.000202]** | **Wald: -4.854** | **<.0001** |  |
| Excluding Esmaillzadeh 2005 (N=33) | Fit statistics | -2LL=-168.1 / AIC=-162.1/BIC=-157.6 |  |  |  |  |
|  | Between study variance (Heterogeneity) | 0.000353 | [0.000228, 0.000619] |  |  | 17.74% |
|  | Intercept | 0.057408 | [0.047300, 0.067515] | Wald: 11.584 | <.0001 |  |
|  | **Whole grain** | **-0.000286** | **[-0.000432, -0.000140]** | **Wald: -3.848** | **0.0001** |  |
| Excluding Parker 2013 (N=31) | Fit statistics | -2LL=-153.6 / AIC=-147.6/BIC=-143.3 |  |  |  |  |
|  | Between study variance (Heterogeneity) | 0.000399 | [0.000254, 0.000719] |  |  | 20.38% |
|  | Intercept | 0.057288 | [0.045449, 0.069126] | Wald: 9.897 | <.0001 |  |
|  | **Whole grain** | **-0.000298** | **[-0.000448, -0.000148]** | **Wald: -3.902** | **0.0001** |  |
| Excluding Wirstrom 2013 (N=34) | Fit statistics | -2LL=-172.8 / AIC=-166.8/BIC=-162.2 |  |  |  |  |
|  | Between study variance (Heterogeneity) | 0.000351 | [0.000227, 0.000614] |  |  | 21.55% |
|  | Intercept | 0.056380 | [0.046541, 0.066219] | Wald: 11.672 | <.0001 |  |
|  | **Whole grain** | **-0.000304** | **[-0.000440, -0.000168]** | **Wald: -4.398** | **<.0001** |  |
